# Nicotine promotes AngII-induced abdominal aortic aortopathies in female and male mice: role of sex hormones

**DOI:** 10.1042/CS20255689

**Published:** 2025-04-23

**Authors:** Mark Ensor, Sean E. Thatcher, Kristen McQuerry, Kory Heier, Heba M. Ali, Victoria English, Lisa A. Cassis, Yasir Alsiraj

**Affiliations:** 1 Department of Pharmacology and Nutritional Sciences, University of Kentucky, Lexington, KY, U.S.A.; 2 Biomedical Education and Data Science, Temple University, Philadelphia, PA, U.S.A.; 3 Department of Biostatistics, University of Kentucky, Lexington, KY, U.S.A.; 4 Department of Pediatrics, University of Kentucky, Lexington, KY, U.S.A.

**Keywords:** abdominal aortic aneurysm, aortopathy, nicotine, sex hormones

## Abstract

Cigarette smoking is a risk factor for abdominal aortic aneurysms (AAAs), with studies suggesting a higher smoking-related AAA risk in women than men. We examined nicotine’s effects on angiotensin II (AngII)-induced AAAs in male and female low-density lipoprotein receptor-deficient (*Ldlr-/-*) mice. Moreover, we defined effects of gonadectomy (GDX) of both sexes on nicotine-induced regulation of AAAs. Male and female *Ldlr-/-* mice (8–12 weeks of age) were infused with AngII with or without nicotine. Mice underwent sham or GDX surgeries prior to infusions of AngII and nicotine. In males, one or both testes were removed. AAA incidence, size, severity, and serum indices of nicotine metabolism were quantified. Effects of testosterone or estrogen on abdominal aortic smooth muscle cells (SMCs) were assessed. Nicotine increased aortic rupture in males, with modest effects in females. GDX reduced AAA incidence in male mice but had modest effects in females. Serum ratios of trans-3-hydroxycotinine to cotinine, an index of nicotine metabolism, were higher in females and increased by GDX in both sexes. Co-infusion of nicotine with AngII increased matrix metalloproteinase 2 (MMP2) mRNA in abdominal aortas of males, but not females. Similarly, testosterone increased MMP2 mRNA in male, but not female abdominal aortic SMCs. Testosterone reduced markers of a contractile SMC phenotype in SMCs from males, with no effects of estrogen in females. In conclusion, nicotine augments AngII-induced AAAs to a greater extent in males, with sex differences related to influences of sex hormones on nicotine metabolism, aortic MMP2 expression, and markers of a contractile SMC phenotype.

## Introduction

Abdominal aortic aneurysms (AAAs) represent a critical health risk characterized by the abnormal dilation of the abdominal aorta, which often remains asymptomatic until reaching a critical size with a high propensity to rupture [1]. AAAs are insidious, potentially leading to severe complications if undetected or untreated. Due to the risk of AAA rupture increasing with size, understanding the risk factors that influence AAA development and progression is crucial.

Cigarette smoking is a major modifiable risk factor for AAA, with recent studies indicating a 1.69 odds ratio for smoking history and AAA development [2]. Smokers are seven times more likely to have an AAA than age-matched non-smokers, with both the duration and the number of cigarettes smoked/day contributing to increased AAA risk [3,4]. Notably, despite marked sex differences in AAA development, with higher prevalence in men than women, smoking is an AAA risk factor in both sexes [5]. Moreover, some studies suggest that smoking is a stronger AAA risk factor in women than in men [6–8].

Nicotine, a principal component of tobacco and a highly addictive alkaloid, has well-documented adverse effects on various cell types involved in AAA development [9]. Results demonstrated that nicotine modulated vascular cell prostacyclin production [10–12], endothelium permeability and production of growth factors [13], endothelial nitric oxide production [14], smooth muscle cell (SMC) growth [15], and arterial stiffness [16]. The degradation of elastin fibers in the medial aorta, through the activation of matrix metalloproteinases (MMPs), has been implicated in nicotine’s influences on AAA formation and progression [17]. In experimental animal models, chronic infusion of nicotine to hyperlipidemic mice promoted AAA formation in male mice [18,19]. Similarly, co-infusions of nicotine with angiotensin II (AngII), an established model of aortic aneurysm formation [20,21], resulted in increased maximal diameters of suprarenal aortas in male mice [22]. These findings suggest that nicotine and AngII independently trigger mechanisms leading to aortic pathology, with a synergistic potentiation observed when both stimuli are combined.

The majority of experimental studies examining effects of nicotine (as a surrogate for smoking) on AAA development have been performed in males. Given the potential for differences in the impact of smoking as a modifiable AAA risk factor between women and men [23], the purpose of this study was to define the impact of sex hormones on nicotine regulation of the AngII-induced AAAs. We assessed the influence of nicotine on the initiation and severity of AngII-induced abdominal AAAs in male and female hypercholesterolemic mice. Additionally, we elucidated the impact of male and female sex hormones on the regulatory effects of nicotine on AngII-induced AAAs in both sexes. Moreover, we investigated mechanisms for sex hormone regulation of pathways involved in nicotine’s influences on aortic SMCs. We hypothesize that nicotine exacerbates AngII-induced AAA development in a sex-dependent manner due to sex hormone-mediated regulation of nicotine pharmacodynamics and/or mechanisms of AAA formation and progression.

## Materials and methods

### Mice

All studies using mice were conducted under approved protocols reviewed by the Institutional Animal Care and Use Committee at the University of Kentucky and conformed to the Guide for the Care and Use of Laboratory Animals published by the NIH. Low-density lipoprotein receptor-deficient (*Ldlr^-/-^*) male and female mice (Stock# 002207, The Jackson Laboratory, Bar Harbor, ME) were used for all studies. Mice were maintained in individually vented cages (five mice/cage) on a light:dark cycle (14:10 hours). Mice were fed Western diet containing 42% kcal from fat (TD88137, Harlan Teklad, Indianapolis, IN) for one week prior to implantation of osmotic minipumps through study endpoint. At the end of each experiment, mice were killed under anesthesia (ketamine/xylazine, 100:10 mg/kg, i.p.), and blood was collected via cardiac puncture.

### AngII and nicotine administration

Anesthetized mice were implanted subcutaneously with osmotic minipumps (Alzet model 1004, Durect Co., Cupertino, CA) containing AngII (Bachem, Torrance, CA) in the absence or presence of nicotine (nicotine bitartrate dehydrate, United States Pharmacopia, Rockville, MD, Cat # 1463304). Preliminary studies demonstrated that co-infusion of nicotine (4 mg/kg/day) with AngII (1,000 ng/kg/min) from the same osmotic minipump resulted in effective nicotine release over 28 days (Supplementary Figure S1). For comparison of females and males, mice were infused with AngII (1,000 ng/kg/min) in the absence or presence of nicotine (4 mg/kg/day) for 28 days. For prolonged infusions to evaluate effects of nicotine on the progression of AAAs, mice were infused with AngII (males, 750 ng/kg/min; females, 1,000 ng/kg/min) plus nicotine (4 mg/kg/day) for 56 days. For this study, new pumps were implanted in anesthetized mice on day 28. For studies in sham or ovariectomized females, mice were infused with AngII (1,000 ng/kg/min) in the absence or presence of nicotine (4 mg/kg/day) for 28 days. For studies in orchiectomized males, mice from each group were infused with AngII (1,000 ng/kg/min) plus nicotine (4 mg/kg/day) for 28 days.

### Orchiectomy

Male *Ldlr^-/-^* mice (8–12 weeks of age) underwent sham surgery or orchiectomy [24] (one or two testes removed) under isoflurane anesthesia (2–3% to effect), with pre- and post-operative analgesics administered (flunixin; 2.5 mg/kg, 24 hours post-surgery). Sham surgery included anesthesia, shaving, sterilization, and incision. For orchiectomized males, a small incision was made, vas deferens were collapsed using a hemostat, and either one or two testes were removed. Vascular supply was ligated via cauterization using a high-temperature fine-tip loop cauterizer. The wound site was monitored for bleeding, and the skin was closed using wound clips (Autoclip stapler). The site was then treated with povidone iodine. All mice underwent a one-week recovery period post-surgery to clear endogenous testicular hormones before the initiation of co-infusions of AngII plus nicotine. Mice were randomly divided into three groups: (1) ‘two testes’, the testes were manipulated but left intact under anesthesia (sham surgery); (2) ‘one testis’, only one testis was removed; (3) ‘no testes’, two testes were removed.

### Ovariectomy

Female mice (8–12 weeks of age) underwent sham surgery or ovariectomy (OVX) [25] under isoflurane anesthesia (2–3%, to effect). To prevent dryness during surgery, ophthalmic ointment (Puralube vet ointment, Dechra) was applied to their eyes. Prior to the procedure, mice received a subcutaneous injection of 2.5 mg/kg flunixin for analgesia, with an additional dose administered 12–16 hours post-surgery. The abdominal region on both flanks was shaved, a depilatory cream (Nair, Inc.) was used to remove hair, and skin was sterilized with povidone iodine and ethanol. For sham surgery, mice underwent anesthesia, sterilization, and incision. For ovariectomized females, an incision (1 cm) was made on each flank to access the fallopian tubes, and the vascular supply to the ovaries was occluded using a hemostat before removing the ovaries. The residual ends of the fallopian tubes were ligated through cauterization. The incision was closed by suturing the peritoneum (5-0 black monofilament nylon suture, Ethilon 1668G) and securing the skin with wound clips (Autoclip stapler). Mice recovered on a heating pad. Two weeks later, after clearance of endogenous ovarian-derived hormones, mice were infused with AngII in the absence or presence of nicotine.

### Ultrasound measurement

Ultrasonography was performed using a Vevo 3100 system with a 40-MHz transducer (FUJIFILM VisualSonics). Mice were anesthetized (isoflurane, 2–3%) and in a supine position to expose the abdominal area. Ultrasound gel was applied to the abdomen to enhance image transmission. The renal arteries were used as anatomical landmarks to locate the abdominal aorta. The renal arteries were identified as paired lateral structures branching from the aorta. Captured images during the systolic phase were used to measure the abdominal lumen diameter. Abdominal aortic lumen diameters of individual mice that completed the entire study duration were included in statistical analysis of lumen diameters.

### Blood pressure measurement

In some studies, systolic and diastolic blood pressure were measured in conscious mice during days 21–25 of AngII infusion using the BP-2000 Visitech blood pressure analysis system. Blood pressure was recorded by the same research personnel at the same time each day for five consecutive days. To allow for acclimation, data from the first two days were excluded from analysis. For the final analysis, blood pressure values from the last three days were averaged, and only data from mice with at least three successful measurements were included in the statistical evaluation.

### Measurement of plasma and serum components

Total serum cholesterol and testosterone concentrations were determined using enzymatic assay kits (Wako Pure Chemical, Richmond, VA, cat#999-02601; Boster, cat#EK7014, respectively). Plasma renin concentration was measured by quantifying AngI generated in the absence or presence of an excess of exogenous rat angiotensinogen (purified from nephrectomized rat plasma) as described previously [26]. Nicotine and metabolites in sera were analyzed using a protein precipitation method and liquid chromatography-tandem mass spectrometry (LC-MS/MS). Sera (20 µl) were spiked with internal standards (0.1 µM nicotine-d_3_ and cotinine-d_3_), mixed, and centrifuged (16 ***g***, 10 min). The supernatant was evaporated to dryness under filtered nitrogen, and the residual pellet was dissolved in mobile phase A for LC-MS/MS analysis. A Thermo Scientific TSQ Altis Plus Triple Quadrupole MS coupled to a Thermo Scientific Vanquish Horizon HPLC system was used for the analysis of target compounds. Chromatographic separation was carried out with a C18 reverse-phase column (Thermo Scientific Accucore RP-MS, 100 mm × 2.1 mm, 2.6 µm) maintained at 40°C, and the flow rate was set to 0.3 ml/min. Mobile phase A consisted of 5 mM ammonium bicarbonate and mobile phase B consisted of 3:1 acetonitrile:methanol. Compounds were separated over a 2.6-minute gradient from 10% mobile phase B to 90% mobile phase B. The MS was equipped with an electrospray ionization source and operated in positive ionization mode. The following instrument settings were used: (1) spray voltage: 2900 V; (2) sheath gas: 50 AU; (3) auxiliary gas: 10 AU; (4) sweep gas: 1 AU; (5) ion transfer tube temperature: 350°C; (6) vaporizer temperature: 320°C. The instrument method was operated in selected reaction monitoring (SRM) mode with a Q1 resolution of 0.7 FWHM and Q3 resolution set to 1.2 FWHM. Quantitative analysis was conducted by monitoring the transitions listed in Supplementary Table S1.

### Quantification of AAAs

AAA incidence was quantified at study endpoint by two observers blinded to the experimental design and included mice that died from confirmed aortic aneurysm rupture post-mortem. To quantify external abdominal aortic diameters of mice completing 28 days of AngII infusions, excised and cleaned tissues were mounted on a black wax background. A Nikon SMZ800 dissecting microscope fitted with a Nikon DS-Ri2 digital camera was used to capture aorta images, and the maximal external diameter of the abdominal aorta was subsequently analyzed.

### Quantification of atherosclerosis

Aortas were stored in 4% paraformaldehyde. After fine cleaning by removing adventitial tissues, the aortic arch was cut open and pinned flat on a black wax, kept moist with saline, and imaged with a Nikon digital camera. Atherosclerotic lesions (% surface area covered by lesions) were traced and quantified using Nikon Imaging System NIS version 5.11 [27].

### Abdominal aortic sectioning, elastin staining, and elastin degradation

Cleaned abdominal aortas with a confirmed AAA from male mice infused with AngII or AngII + nicotine were paraffin embedded and sectioned (5 µm). Three pairs of aortic sections were placed on a total of five slides. Aortic sections, from different locations of the artery, covering a total of 50 µm were used for histological analysis. Aortic sections were deparaffinized and rehydrated through a series of graded alcohols and distilled water and stained with Van Gieson to visualize collagen and elastin fibers in the aortic wall. Six sections (50 µm apart) from male abdominal aortas infused with AngII or AngII + nicotine were used to quantify the number of elastin breaks per section.

### Murine aortic SMCs

Male or female mice were used to isolate abdominal aortic vascular smooth muscle cells (VSMCs). VSMCs were isolated and plated in six-well plates at a seeding density of 300,000 cells/well in DMEM/F12 + 10% charcoal stripped fetal bovine serum. The cells were incubated at 37°C for 24 hours before treatment (72 hours) with either 17-β estradiol (100 nM; female cells) or testosterone (100 nM; male cells). RNA was isolated from cells following the procedure for the Promega Maxwell RSC system using the simply RNA Tissue Kit (REF# AS1340, Promega, Madison, WI), and RNA concentration was quantified on a Nanodrop 2000. cDNA was synthesized (400 ng of RNA/reaction) using qScript™ cDNA SuperMix (Quanta Biosciences, cat# 95048-500, Gaithersburg, MD), using reaction conditions of 25°C for 5 minutes, followed by 42°C for 30 minutes and 85°C for 5 minutes. Primer sequences were as follows:

**Table:** 

Gene	Forward	Reverse
α-2nAchR	5′-AACCTGCTCTGAACTGTCCTGTGT-3′	5′-AATGTTCTGAAAGGCCCAATCCGC-3′
α-7nAchR	5′-ACCTGCAGATGCAAGAGGCAGATA-3′	5′-AGGAATGAGCAGGTTGAGGCCATA-3′
MMP2	5′-CCAGCAAGTAGATGCTGCCT-3′	5′-GGGGTCCATTTTCTTCTTCA-3′
Sm22alpha	5′-GATATGGCAGCAGTGCAGAG-3′	5′-AGTTGGCTGTCTGTGAAGTC-3′
Calponin	5′-GCGTCACCTCTATGATCCCAA-3′	5′-CCCAGACCTGGCTCAAAGAT-3′
ACTA2	5′-TCCTGACGCTGAAGTATCCGAT-3′	5′-GGCCACACGAAGCTCGTTATAG-3′
β-actin	5′-TGAGCTGCGTTTTACACCCT-3′	5′-GCCTTCACCGTTCCAGTTTT-3′
GAPDH	5′-GCCAAAAGGGTCATCATCTC-3′	5′-GGCCATCCACAGTCTTCT-3′
18S	5′-CGGCTACCACATCCAAGGAA-3′	5′-GCTGGAATTACCGCGGCT-3′

Gene expression was quantified by RT-PCR using PerfeCTa^®^ SYBR^®^ Green FastMix^®^ (Quanta Biosciences, cat# 95071-012, Gaithersburg, MD) on a BioRad quantitative real-time PCR thermocycler (CF96 Real-Time system, BioRad, Hercules, CA). mRNA abundance was determined using the ΔΔCt method and normalized to β-actin, GAPDH, and/or 18S.

### Statistical analyses

Data are illustrated as mean ± SEM. Means of normally distributed data were analyzed using unpaired Student *t*-tests for two groups. For experiments with more than two groups and/or treatments, one- or two-way ANOVAs were used for analysis, followed by pairwise comparison test for significance. Two-way repeated-measures ANOVA over time was used for the analysis of longitudinal data of abdominal aortic lumen diameters with between-group factors of sex or treatment. If differences existed between groups, a Holm–Sidak post hoc analysis was performed. Incidence of AAAs was analyzed by Fisher’s exact test when two groups were examined. Survival rates were analyzed using log-rank test. Statistical analysis was performed using GraphPad Prism 8 and SAS version 9.4. *P*≤0.05 was considered significant.

## Results

### Nicotine augments the formation and severity of AngII-induced aortopathies in males, with modest effects in females

We investigated the effects of nicotine on AngII-induced AAAs in female and male *Ldlr^-/-^* mice. While body weights were increased significantly in males compared with females (both treatment groups, Table 1, AngII ± nicotine in female and male mice; *P*<0.05), nicotine had no significant effect on body weight in male or female mice (Table 1, AngII + nicotine groups within each sex). Systolic and diastolic blood pressures were measured during the third week of AngII infusion. Nicotine co-infusion did not significantly alter systolic or diastolic blood pressure in male or female mice (Supplementary Figure S2). Sera triglyceride concentrations were not significantly different between males and females (AngII groups; *P*>0.05). However, nicotine co-infusion increased sera triglyceride concentrations in male, but not female mice (Table 1; *P*<0.05 compared with male AngII). Total serum cholesterol concentrations were higher in males than females (both treatment groups, Table 1, *P*<0.05 compared with female), with no significant influences of co-infusions of AngII + nicotine in either sex. Plasma renin concentrations were not different between treatment groups or sexes (Table 1; *P*>0.05). Co-infusion of nicotine with AngII reduced the percent atherosclerotic lesion surface areas of aortic arches of female, but not significantly in male mice (Table 1, *P*<0.05 female AngII + nicotine compared with female AngII). In both male and female mice, infusions of nicotine resulted in increased sera concentrations of nicotine and cotinine compared with AngII within sex, with no significant differences in sera levels of nicotine and/or cotinine between sexes (Table 1, *P*<0.05 compared with AngII only within sex).

**Table 1:** Characteristics of female and male mice infused with AngII ± nicotine under different experimental conditions

**AngII ± nicotine in female and male mice**				
**Parameters**	**Female AngII**	**Female AngII + Nic**	**Male AngII**	**Male AngII + Nic**
Body weight (g)	21.5 ± 1.2	21.7 ± 1.4	26.3 ± 2.3*^1^	25.7 ± 1.6*^1^
Serum triglyceride (mg/dl)	558 ± 77	662 ± 57	692 ± 51	940 ± 69*^1^, #^2^
Total serum cholesterol (mg/dl)	1472 ± 117	1624 ± 125	1965 ± 147*^1^	1978 ± 175
% Atherosclerotic lesion in aortic arch	12.95 ± 0.88	7.52 ± 1.05#^2^	11.4 ± 1.74	8.06 ± 1.2
Plasma renin concentration (ng/ml)	3.7 ± 0.13	4.71 ± 0.39	3.82 ± 0.16	4.66 ± 0.39
Nicotine (ng/ml)	0.0 ± 0.0	24.41 ± 3.25#^2^	0.0 ± 0.0	22.85 ± 2.9#^2^
Cotinine (ng/ml)	0.0 ± 0.0	25.23 ± 2.69#^2^	0.0 ± 0.0	43.94 ± 3.39*^1^,#^2^
**Prolonged exposures of AngII ± nicotine in females and males**				
**Parameters**	**Female AngII**	**Female AngII + Nic**	**Male AngII**	**Male AngII + Nic**
Body weight (g)	21.93 ± 0.52	21.71 ± 0.52	29.34 ± 1.09*^1^	27.58 ± 0.9*^1^
Serum triglyceride (mg/dl)	577 ± 398	346 ± 181	457 ± 342	550 ± 305
Total serum cholesterol (mg/dl)	2128 ± 580	1177 ± 429	1601 ± 883	1848 ± 1018
% Atherosclerotic lesion in aortic arch	22 ± 7.9	13 ± 3.9#^2^	17.92 ± 7.87	20.53 ± 8.08
Plasma renin concentration (ng/ml)	2.86 ± 1.2	2.38 ± 0.22	3.52 ± 0.89	2.98 ± 0.22
Nicotine (ng/ml)	1.2 ± 1.17	73.8 ± 27.01#^2^	5.4 ± 2.03	103.04 ± 63.69#^2^
Cotinine (ng/ml)	5.18 ± 3.72	27.24 ± 7.73#^2^	2.8 ± 0.38	61.21 ± 8.6*^1^,#^2^
**Effects of OVX in AngII ± nicotine females**				
**Parameters**	**Sham AngII**	**Sham AngII + Nic**	**OVX AngII**	**OVX AngII + Nic**
Body weight (g)	22 ± 0.44	22.1 ± 0.45	23.8 ± 0.58	22.7 ± 0.55
% Atherosclerotic lesion in aortic arch	10.07 ± 2.69	11.96 ± 2.56	14.49 ± 2.03	13.80 ± 1.43
Plasma renin concentration (ng/ml)	2.99 ± 0.76	3.9 ± 0.97	2.19 ± 0.06	2.44 ± 0.10
Nicotine (ng/ml)	1.2 ± 0.195	36.87 ± 12.4#^2^	1.22 ± 0.275	29.94 ± 6.3#^2^
Cotinine (ng/ml)	3.43 ± 0.254	43.1 ± 7.64#^2^	4.0 ± 0.348	51.45 ± 7.08#^2^
**Effect of ORCH (one or both testes) in AngII + nicotine males**				
**Parameters**	**Two testis AngII + Nic**	**One testis AngII + Nic**	**No testis AngII + Nic**	
Body weight (g)	28.8 ± 1.81	25.4 ± 0.92	28.1 ± 0.52	
Serum testosterone (ng/ml)	0.206 ± 0.06	0.33 ± 0.146	0.053 ± 0.029	
Seminal vesicles (g)	0.256 ± 0.052	0.225 ± 0.045	0.005 ± 0.012	
Nicotine (ng/ml)	23.05 ± 14.55	43.42 ± 23.02	14.09 ± 4.54	
Cotinine (ng/ml)	44.87 ± 4.99	63.60 ± 14.39	49.25 ± 16.29	

1 *, *P*<0.05 compared with females within treatment.

2 #, *P*<0.05 compared with AngII within sex.

**Figure 1:**
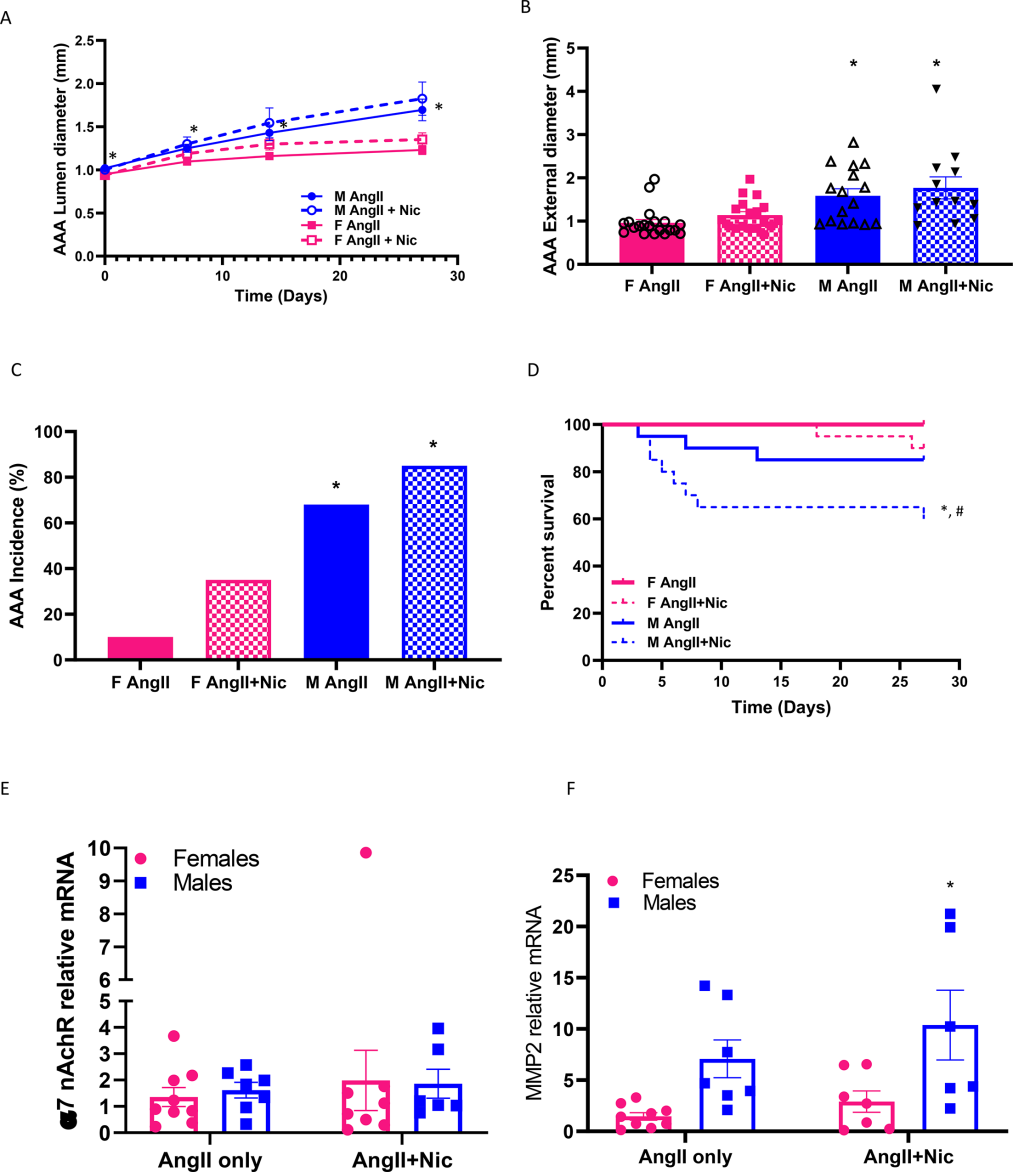
**Effects of nicotine on AngII-induced AAAs in female and male mice**. Female and male mice were infused with AngII in the absence or presence of nicotine for 28 days. (**A**) Abdominal aortic internal lumen diameters measured by ultrasound at different times of infusions. *, *P*<0.05 compared with day 0. (**B**) Maximal AAA external diameters at study endpoint. *, *P*<0.05 compared with female within treatment. (**C**) AAA incidence. *, *P*<0.05 compared with female within treatment. (**D**) Percent survival of mice in each group (aortic rupture confirmed post-mortem). *, *P*<0.05 compared with female within treatment group. #, *P*<0.05 compared with AngII within sex. (**E**) Abdominal aortic α7nAchR mRNA abundance. (**F**) Abdominal aortic MMP2 mRNA abundance. *, *P*<0.05 compared with AngII only within sex. Symbols represent individual values (**E,F**), while bars are mean ± SEM from F AngII *N* = 20, F AngII + Nic *N* = 17, M AngII *N* = 16, M AngII + Nic *N* = 12 in A&B; F AngII *N* = 20, F AngII + Nic *N* = 20, M AngII *N* = 19, M AngII + Nic *N* = 20 in C&D; and F AngII *N* = 9, F AngII + Nic *N* = 8, M AngII *N* = 7, M AngII + Nic *N* = 6 in E&F. AAAs, abdominal aortic aneurysms; AngII, angiotensin II.

In mice surviving the 28-day infusion protocol, there were no significant differences in abdominal aortic lumen diameters at baseline (day 0) or at each timepoint measured between AngII versus AngII + nicotine within each sex (Figure 1A, *P*>0.05 compared with AngII). However, male mice (AngII, AngII + nicotine) exhibited an increase in abdominal aortic lumen diameter over time (Figure 1A, *P*<0.05), with no significant effect of AngII or AngII + nicotine on abdominal aortic lumen diameters in females (Figure 1A, *P*>0.05). Similarly, at study endpoint, maximal external AAA diameters of mice surviving the 28 days of infusions were higher in males than females (Figure 1B, AngII, *P*<0.05), but there was no significant effect of nicotine in males or females (*P*>0.05). AAA incidence, which includes mice experiencing post-mortem confirmed aortic rupture, increased from 10 to 35% in female mice co-infused with AngII + nicotine, and from 70 to 90% in males co-infused with AngII + nicotine (Figure 1C). Moreover, AAA incidence was greater in male than female mice of each treatment group (Figure 1C, *P*<0.05 compared with female within treatment). Percent survival was decreased significantly in male mice co-infused with AngII + nicotine compared with AngII (Figure 1D, *P*<0.05), with no influence of nicotine on percent survival of females.

Using abdominal aortas harvested for RNA extraction and RT-PCR at study endpoint, mRNA abundance of the α-7 nicotinic acetylcholine (α-7nACh) receptor, implicated in the regulation of nicotine’s influences on the vasculature [28], was not different between females or males (AngII), or influenced by co-infusions of AngII + nicotine (Figure 1E). In contrast, mRNA abundance of MMP2, implicated in the vascular effects of nicotine [29], was increased significantly in abdominal aortas from male compared with female mice (Figure 1F, AngII only; *P*<0.05). Moreover, MMP2 mRNA abundance was increased significantly by co-infusions of AngII + nicotine in males, but not in abdominal aortas from females (Figure 1F; *P*<0.05 compared with AngII only for males).

### Prolonged nicotine exposures augment aortic ruptures of AngII-infused male, but not female mice

The studies described above demonstrate the effects of nicotine to promote aortic rupture and decrease percent survival in male AngII-infused mice, indicating influences of nicotine on the development and severity of AAAs. To define the effects of nicotine on the progressive growth of AAAs, we extended the durations of nicotine + AngII exposures. Due to high aortic rupture rates of males co-infused with AngII (1,000 ng/kg/min) plus nicotine for 28 days (Figure 1D), we lowered the AngII infusion dose (750 ng/kg/min) in males and extended durations of infusions (56 days) in both sexes. Body weights were higher in males than female mice of both treatment groups, with no significant effects of nicotine on body weight in either sex (Table 1, prolonged exposures of AngII ± nicotine in females and males; *P*<0.05 compared with female within treatment). Nicotine co-infusions had no significant effect on sera triglyceride concentrations, total sera cholesterol concentrations, or plasma renin concentrations in either sex (Table 1). The percent surface atherosclerotic lesion area of the aortic arch was decreased by co-infusion of AngII + nicotine in female, but not male mice (Table 1; *P*<0.05 compared with female AngII). Upon more prolonged exposures, sera nicotine and cotinine levels were increased in both sexes compared with 28 days of co-infusions (Table 1). Moreover, sera cotinine concentrations were significantly lower in female than male mice co-infused with AngII + nicotine (Table 1; *P*<0.05 compared with female AngII + nicotine).

**Figure 2:**
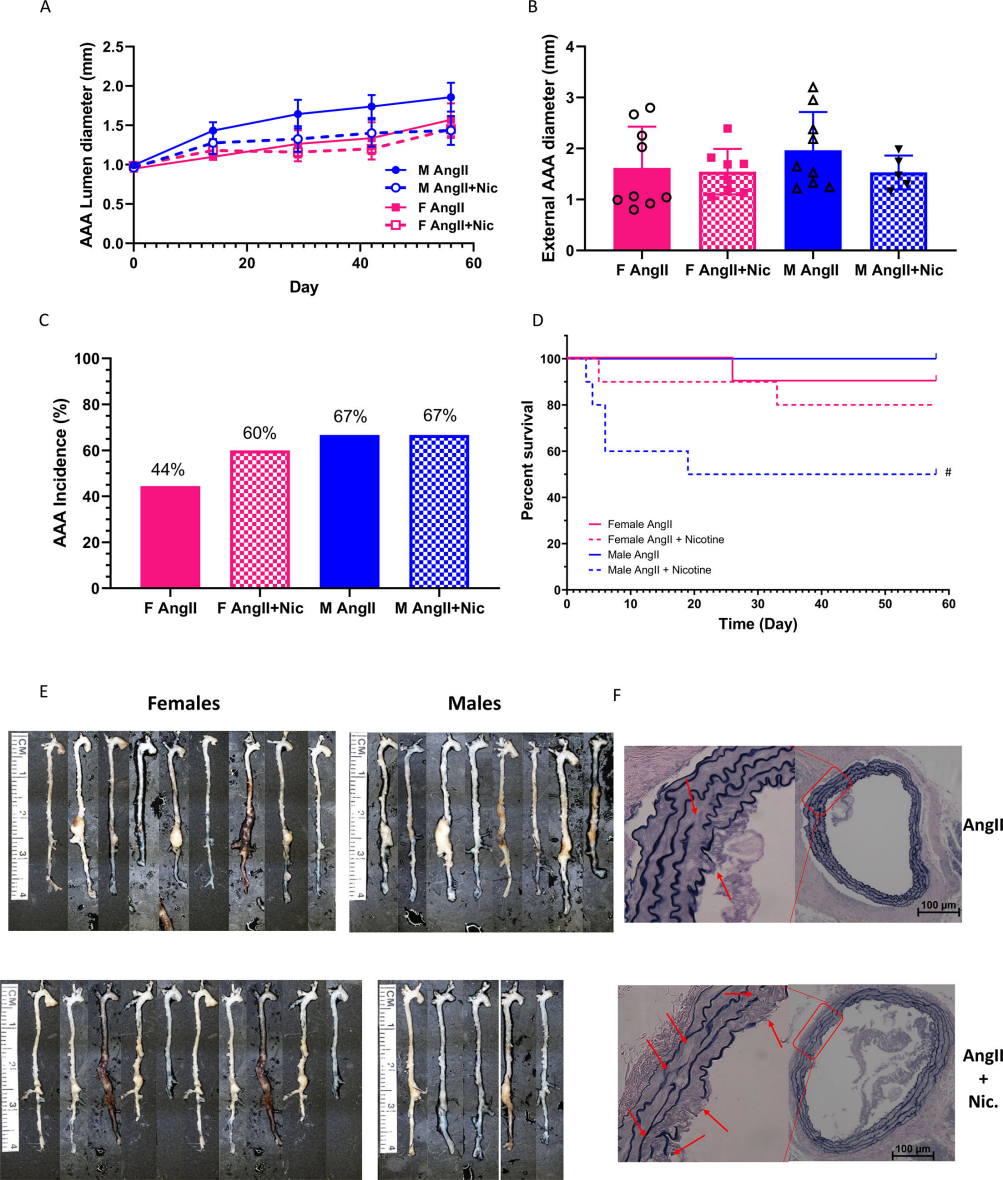
**Effects of prolonged exposures to nicotine on AngII-induced AAAs of female and male mice**. Female (1,000 ng/kg/min AngII) or male mice (750 ng/kg/min AngII) infused with AngII in the absence or presence of nicotine for 56 days. (**A**) Abdominal aortic internal lumen diameters measured by ultrasound at different times of infusions. (**B**) Maximal AAA external diameters at study endpoint. (**C**) AAA incidence. (**D**) Percent survival of mice in each group (aortic rupture confirmed post-mortem). #, *P*<0.05 compared with AngII within sex. (**E**) Cleaned aortas from mice of each group. (**F**) Abdominal aortic tissue sections stained with Van Gieson’s. Arrows denote breaks in medial elastin. Symbols represent individual mice, while bars are mean ± SEM from F AngII *N* = 9, F AngII + Nic *N* = 8, M AngII *N* = 9, M AngII + Nic *N* = 5 in A&B; F AngII *N* = 9, F AngII + Nic *N* = 10, M AngII *N* = 9, M AngII + Nic *N* = 9 in C&D. AAAs, abdominal aortic aneurysms; AngII, angiotensin II.

Prolonged infusions of AngII to males at a lower dose of 750 ng/kg/min increased abdominal aortic lumen diameters compared with day 0 (baseline), with no differences in baseline or effects of AngII (with or without nicotine) on abdominal aortic lumen diameters of females (1,000 ng/kg/min) (Figure 2A; *P*>0.05 compared with day 0 for males). Moreover, co-infusions of AngII + nicotine had no effect on abdominal aortic lumen diameters (Figure 2A; *P*>0.05), maximal AA external diameters (Figure 2B; *P*>0.05), or AAA incidence (Figure 2C; *P*>0.05) in either sex. However, prolonged exposures of male mice co-infused with AngII + nicotine led to a pronounced reduction in percent survival, leading to death associated with aortic rupture in 60% of male mice (Figure 2D and E; *P*<0.05 compared with AngII for males). Abdominal aortic tissue sections from male mice co-infused with AngII + nicotine exhibited significantly greater numbers of medial elastin breaks compared with intact medial elastin in aortic sections from male AngII-infused mice (Figure 2F; Supplementary Figure S3).

### OVX of female mice has no significant effects on nicotine plus AngII-induced AAAs

Previous studies demonstrated that OVX of female apolipoprotein-E-deficient mice had no significant effect on the formation of AngII-induced AAAs [25] but did influence AAA progression [30]. Despite suggested higher AAA smoking risk in women than men [23], it remains unclear whether sex hormones influence nicotine’s effects on AAA development or severity. Therefore, we used OVX as a mode of eliminating female ovarian sex hormones in studies of nicotine’s influences on AngII-induced AAAs. Body weight, plasma renin concentrations, and the percent atherosclerotic lesions in the aortic arch were not different between groups at study endpoint (Table 1, effects of OVX on AngII ± nicotine females). Sera concentrations of nicotine and cotinine were increased in mice co-infused with AngII + nicotine compared with AngII, with no differences in nicotine or cotinine levels between sham versus OVX females (Table 1, AngII + nicotine groups).

**Figure 3:**
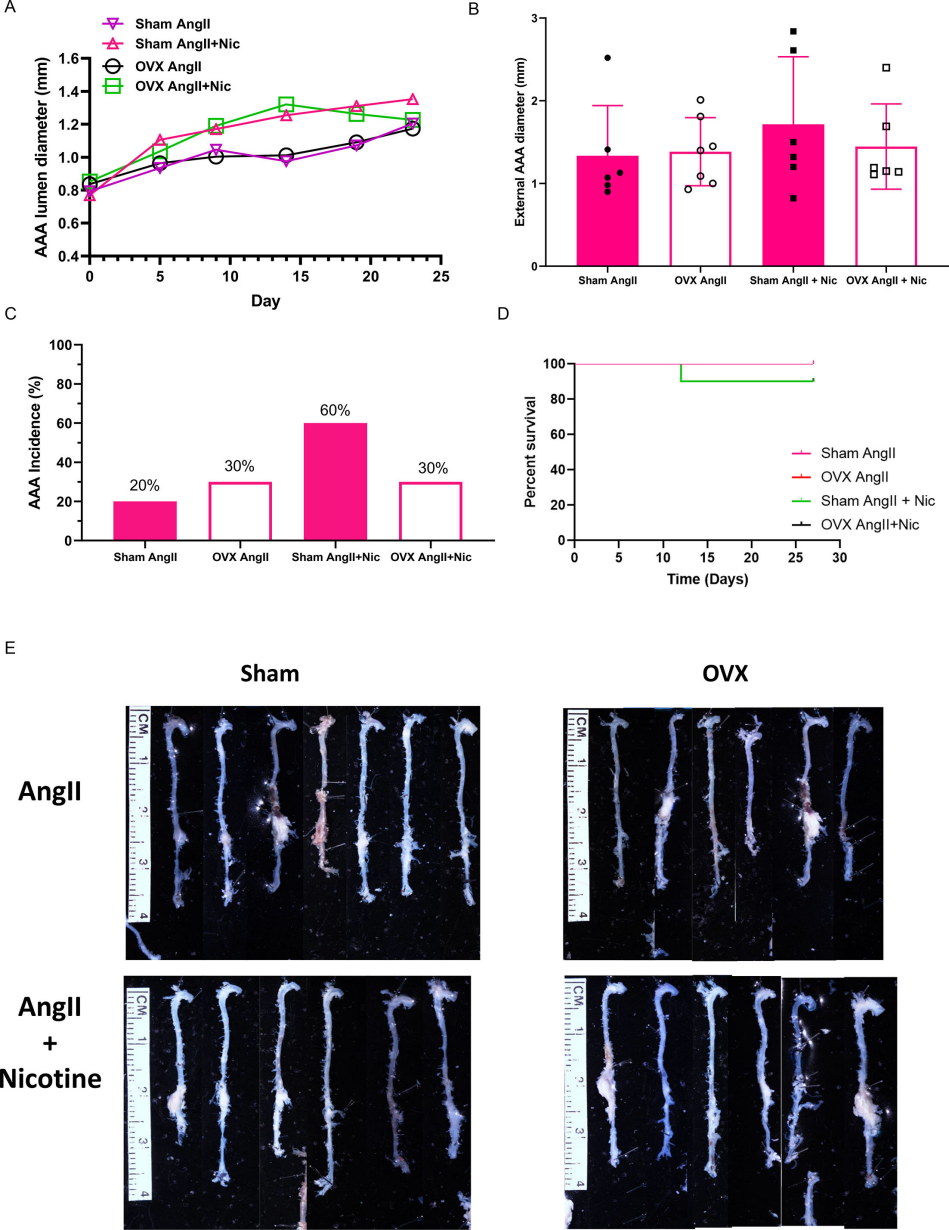
**Effects of ovariectomy (OVX) on nicotine regulation of AngII-induced AAAs in female mice**. Female mice were sham-operated or OVX two weeks prior to infusions of AngII ± nicotine. (**A**) Abdominal aortic internal lumen diameters measured by ultrasound at different times of infusions from sham AngII *N* = 10, sham AngII + Nic *N* = 9, OVX AngII *N* = 10, OVX AngII + Nic *N* = 9. (**B**) Maximal AAA external diameters at study endpoint from sham AngII *N* = 6, sham AngII + Nic *N* = 6, OVX AngII *N* = 7, OVX AngII + Nic *N* = 6. (**C**) AAA incidence. (**D**) Percent survival of mice in each group (aortic rupture confirmed post-mortem). (**E**) Cleaned aortas from mice of each group. Symbols represent individual mice, while bars are mean ± SEM. AAAs, abdominal aortic aneurysms; AngII, angiotensin II.

Abdominal aortic lumen diameters over time of infusion (Figure 3A) and maximal AAA external diameters at study endpoint (Figure 3B) were not different between groups. AAA incidence increased from 20 to 60% (*P*>0.05) in sham female mice infused with Ang + nicotine compared with AngII (Figure 3C). In contrast, AAA incidence decreased from 60 to 30% (*P*>0.05) in OVX AngII + nicotine compared with sham AngII + nicotine-infused females, but this reduction was not significantly different (Figure 3C). There was no significant effect of OVX or nicotine on percent survival of AngII-induced females (Figure 3D). Representative aortas from mice of each group are illustrated in Figure 3E.

### Removal of male testes decreases nicotine’s effects on AngII-induced AAAs

Previous studies demonstrated that testosterone augments AngII-induced AAAs in both male and female mice [24,25,31–34]. However, it remains unclear whether testosterone influences the ability of nicotine to augment the severity of AngII-induced AAAs in male mice. To define the interactions between testosterone and AngII-induced AAAs in males, we removed either one or both testes prior to co-infusions of AngII + nicotine. Body weight was not significantly different between treatment groups (Table 1, effect of ORCH; one or both testes in AngII + nicotine males). Sera testosterone concentrations and seminal vesicle weights were not significantly different between mice with two or one testes (Table 1; *P*>0.05). Sera concentrations of nicotine and cotinine were not significantly different between groups (Table 1; *P*>0.05).

**Figure 4:**
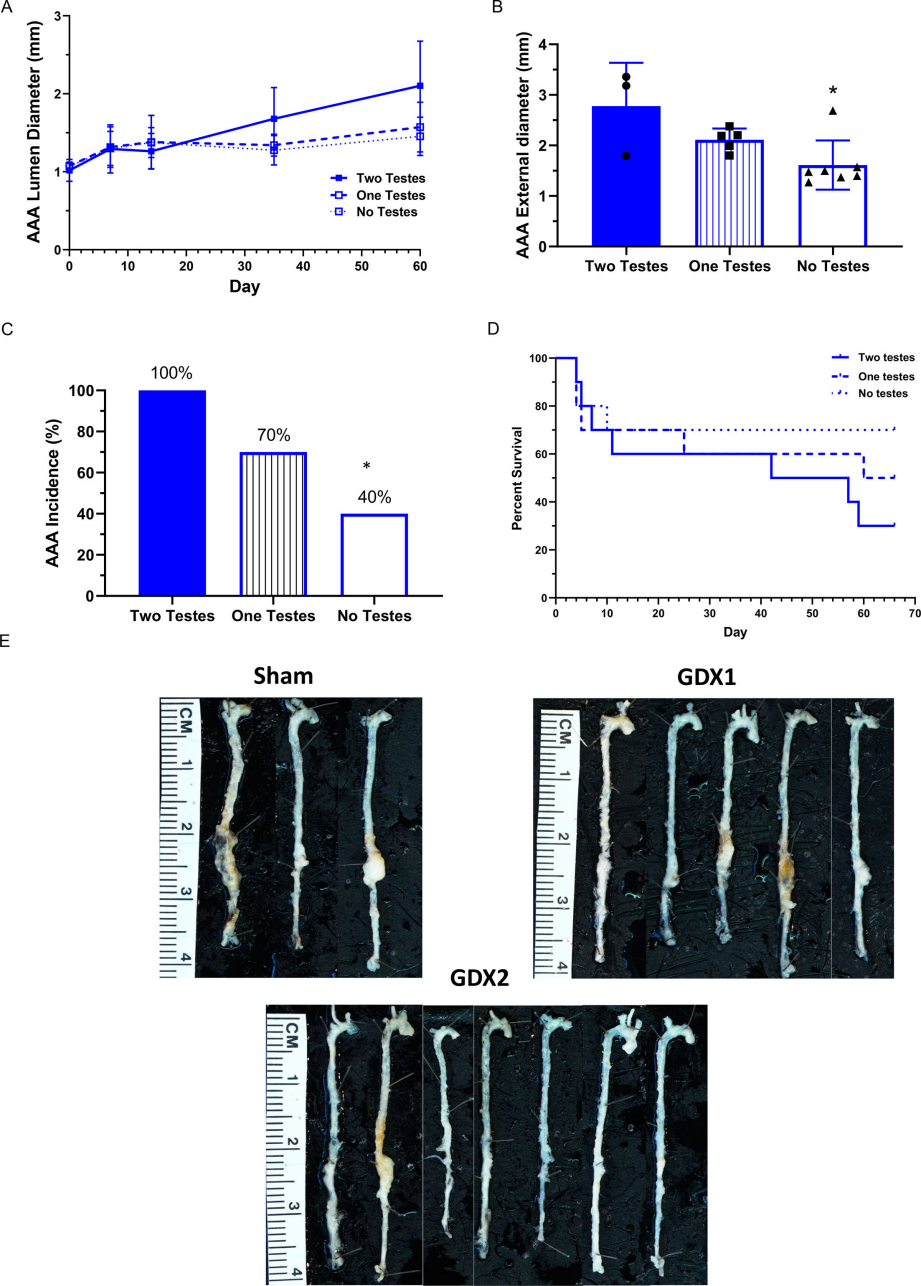
**Effects of orchiectomy on nicotine regulation of AngII-induced AAAs in male mice**. Male mice were sham-operated (two testes) or had one (one teste) or two testes removed (no testes) one week prior to co-infusions of AngII plus nicotine. (**A**) Abdominal aortic internal lumen diameters measured by ultrasound at different times of infusions from two testes *N* = 3, one testes *N* = 5, no testes *N* = 7. (**B**) Maximal AAA external diameters at study endpoint from two testes *N* = 3, one testis *N* = 5, no testes *N* = 7. *, *P*<0.05 compared with two testes. (**C**) AAA incidence. *, *P*<0.05 compared with two testes. (**D**) Percent survival of mice in each group (aortic rupture confirmed post-mortem). (**E**) Cleaned aortas from mice of each group. Symbols represent individual mice, while bars are mean ± SEM. AAAs, abdominal aortic aneurysms; AngII, angiotensin II.

Abdominal aortic lumen diameters were not significantly different at individual time points examined between groups (Figure 4A; *P*>0.05). Maximal AAA external diameters were decreased significantly in AngII + nicotine co-infused mice with no testes compared with mice with two testes (Figure 4B; *P*<0.05). Similarly, AAA incidence was decreased significantly (from 100 to 40%) in AngII + nicotine co-infused mice with no testes compared with mice with two testes (Figure 4C; *P*<0.05). Percent survival was lowest in mice with two testes compared with other groups (Figure 4D). The low number of AngII + nicotine co-infused mice with two testes surviving the experimental protocol (Figure 4E, aortas from mice in each treatment group) prohibited the quantification of percent atherosclerotic lesion surface area.

### Females exhibit higher sera trans-3-hydroxycotinine/cotinine ratios indicative of faster rates of nicotine metabolism compared with males

**Figure 5:**
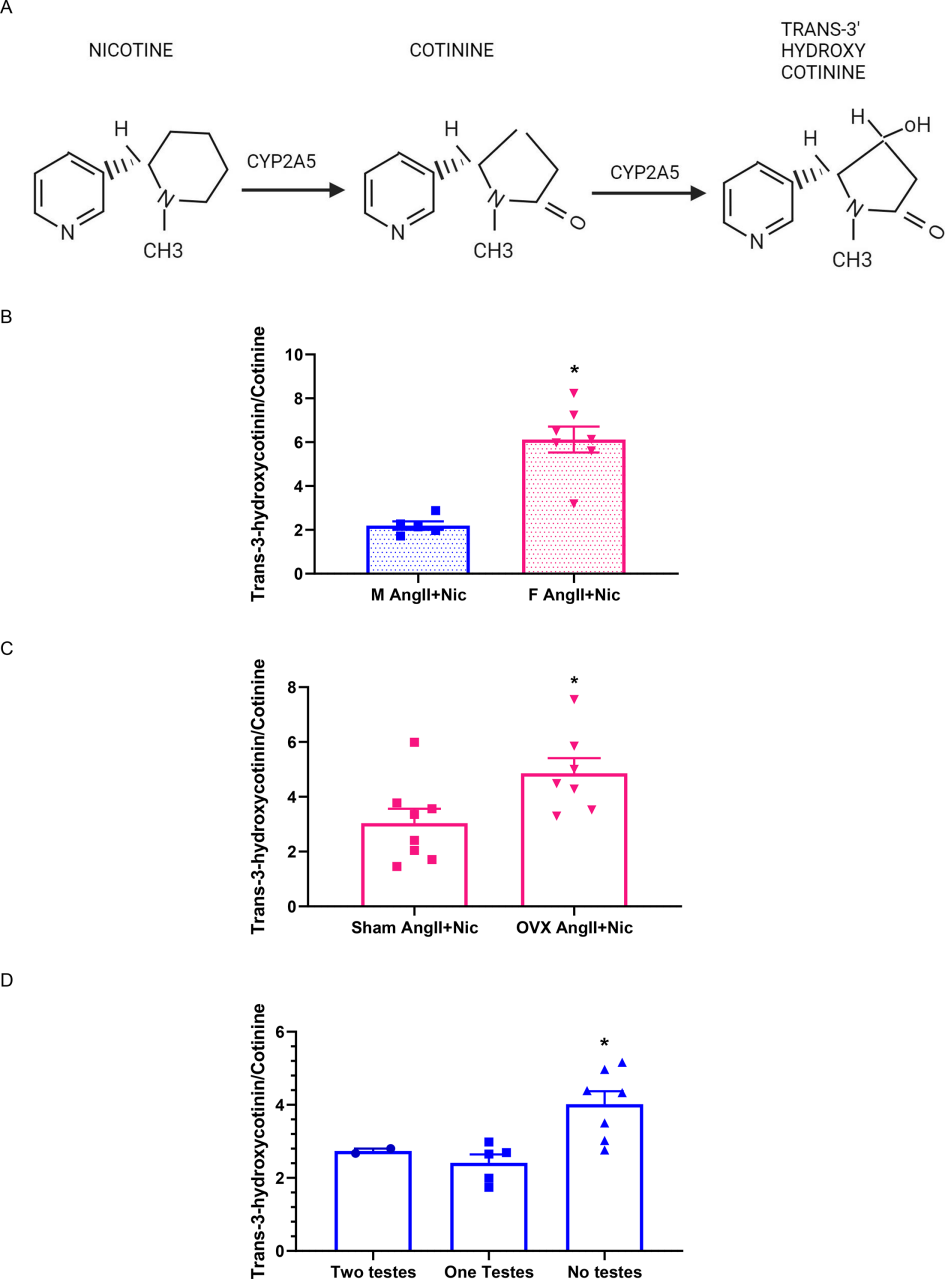
**Sera levels of trans-3-hydroxycotinine/cotinine as an index of nicotine metabolism**. Sera levels of nicotine metabolites, trans-3-hydroxycotinine and cotinine, and ratios of these metabolites, measured using LC-MS/MS. (**A**) Nicotine metabolism pathway. (**B**) Sera trans-3-hydroxycotinine/cotinine ratios in female and male mice co-infused with AngII plus nicotine for 56 days. *, *P*<0.05 compared with males. (**C**) Sera trans-3-hydroxycotinine/cotinine ratios in sham and ovariectomy females co-infused with AngII plus nicotine for 28 days. *, *P*<0.05 compared with sham. (**D**) Sera trans-3-hydroxycotinine/cotinine ratios in male mice with removal of one or both testes. *, *P*<0.05 compared with two testes. Symbols represent individual mice, while bars are mean ± SEM from *N* = 2–8 mice/group.

Nicotine is enzymatically metabolized to cotinine and subsequently to trans-3'-hydroxycotinine with the levels of the parent and metabolized forms of nicotine present in the circulation [35] (Figure 5A; Table 1). Cotinine levels in the circulation serve as a biomarker for quantifying tobacco consumption in active smokers and assessing exposure to secondhand smoke in non-smokers [36,37]. The ratio of the nicotine metabolite, trans-3′-hydroxycotinine, to its substrate, cotinine, reflects the metabolic rate of nicotine [38]. MS analysis enabled quantification of sera levels of nicotine, cotinine (Table 1), and trans-3-hydroxycotinine (Supplementary Figure S4). We examined ratios of trans-3-hydroxycotinine to cotinine levels in sera as an index of nicotine metabolism across each study (Figure 5B–D). Female AngII + nicotine co-infused mice (56 days of infusions) had higher sera trans-3-hydroxycotinine/cotinine ratios than males (Figure 5B; *P*<0.05). Moreover, sera trans-3-hydroxycotinine/cotinine ratios were significantly higher in OVX compared with sham females co-infused with AngII + nicotine (28 days of infusions) (Figure 5C; *P*<0.05). Similarly, sera trans-3-hydroxycotinine/cotinine ratios were significantly higher in male AngII + nicotine co-infused mice with no testes compared with mice with two testes (28 days of infusions) (Figure 5D; *P*<0.05).

### The influence of sex hormones on murine abdominal aortic VSMC from both sexes

**Figure 6:**
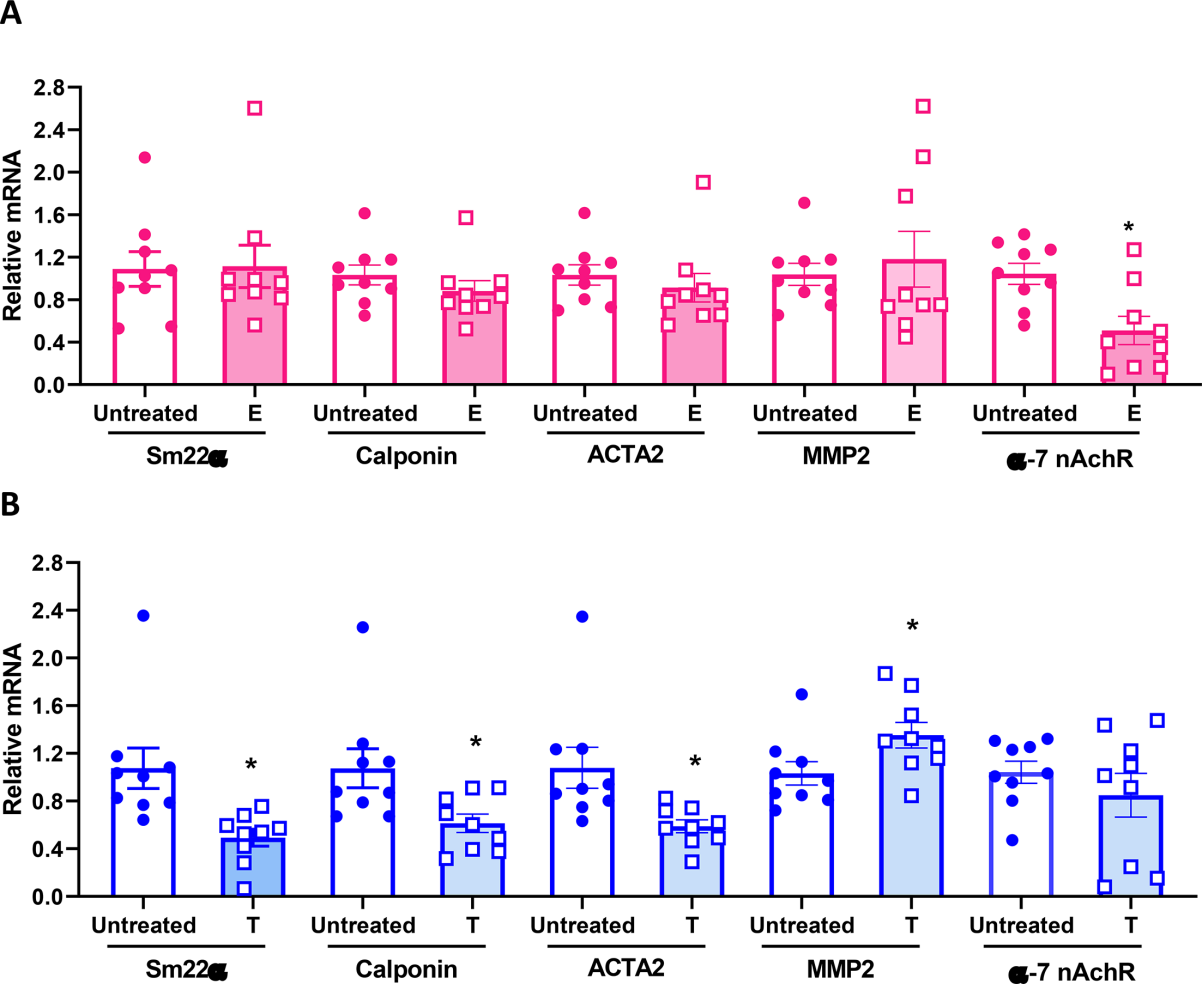
**Sex hormone regulation in abdominal aortic VSMC**. Abdominal aortic female and male VSMC were treated with 17-β-estradiol or testosterone, respectively. (**A**) mRNA abundance of Sm22α, Calponin, ACTA2, MMP2, and α-7AchR in female VSMC. *P<0.05 compared with untreated. (**B**) mRNA abundance of Sm22α, Calponin, ACTA2, MMP2, and α-7AchR in male VSMC. *P<0.05 compared with untreated. Symbols represent individual values, while bars are mean ± SEM from *N* = 9/group/treatment. VSMC, vascular smooth muscle cell.

The abovementioned results indicate more pronounced influences of nicotine on AAA severity in males than females, which were ameliorated upon removal of male testosterone. Given the established literature indicating protective effects of estrogen on the cardiovascular system [39–43], it was surprising that the removal of female sex hormones by OVX had no significant effects on nicotine regulation of AngII-induced AAAs (Figure 3C). Moreover, rather than promoting nicotine regulation of AngII-induced AAAs as might be expected with protective influences of estrogen, OVX modestly reduced nicotine’s effects, although results were not statistically significant. These findings may be influenced by the effects of sex hormones on nicotine metabolism, where gonadectomy (GDX) of both sexes increased an index of nicotine metabolism (Figure 5C and D). Nicotine has several reported effects on VSMC such as degradation of elastin (see Figure 2F) through stimulation of matrix MMPs, including SMC-derived MMP2 [44]. To identify cell-based mechanisms of sex hormones that influence responses to nicotine, abdominal aortic VSMCs harvested from adult male and female mice were treated with testosterone or 17-β-estradiol, respectively, and harvested for quantification of α-7nACh receptor or MMP2 mRNA abundance by RT-PCR. We chose α-7nAch receptor because of its presence in SMC [45] and potential regulation by female sex hormones [46]. This nicotinic receptor subtype has also been implicated as protective against AAA formation [47]. Using charcoal-stripped sera to remove sex hormones, incubation with 17-β-estradiol (100 nM; Figure 6A, female sex hormone) resulted in a significant reduction in α-7nAch receptor mRNA abundance (*P*<0.05 compared with untreated). We also examined another nicotinic receptor, the α-2nAch receptor, but found no significant differences between sexes or treatment groups (Supplementary Figure S5). Moreover, the incubation of male VSMC with testosterone, but not female VSMC incubated with 17-β-estradiol, resulted in a significant increase in MMP2 mRNA abundance (Figure 6; *P*<0.05 compared with untreated male VSMCs).

SMCs have the capability to switch from a contractile to a synthetic phenotype to adapt to mechanical stress [48]. Imbalance in the switch of SMCs from a contractile to a synthetic phenotype has been suggested to contribute to several cardiovascular diseases, including AAAs [49]. Synthetic SMCs are proliferative, while contractile SMCs are differentiated and express specific contractile proteins [50]. We defined the effects of female (100 nM; 17-β-estradiol) or male (100 nM; testosterone) sex hormones on markers of a contractile abdominal aortic VSMC phenotype. The incubation of female VSMCs with 17-β-estradiol had no effect on mRNA abundance of contractile markers such as SM22α, calponin, and ACTA2 (Figure 6A; *P*>0.05 compared with untreated). In contrast, the incubation of male SMCs with testosterone resulted in a significant reduction in mRNA abundance of SM22α, calponin, and ACTA2 (Figure 6B; *P*<0.05 compared with untreated), indicative of a potential switch to a synthetic SMC phenotype.

## Discussion

There is an irrefutable evidence that smoking is a strong, modifiable risk factor for AAA development [51–53]. However, while smoking is a risk factor for AAAs in both women and men [5], some studies suggest smoking as a stronger risk factor in women [6–8]. Results from this study, using nicotine as a surrogate for smoking in male and female mice infused with AngII, provide significant insights into a complex interplay between nicotine, sex hormones, and AngII-induced AAAs in male and female mice. Our approach incorporated exposures of both sexes of AngII-infused mice to nicotine, since the majority of previous experimental studies studying the impact of smoking and/or nicotine on AAA development were performed in males. Moreover, we used GDX to study the influence of sex hormones on nicotine’s impact on AAA development and progression, coupled with cell-based studies examining mechanisms of nicotine’s effects between sexes. This study is the first to simultaneously evaluate sex-specific effects of nicotine and the underlying mechanisms driven by sex hormones, such as the regulation of SMC phenotypes and MMP activity.

The present study provides several key findings: (1) nicotine augments the severity of AngII-induced AAAs in males, with modest effects in females; (2) upon prolonged exposures to AngII + nicotine to more closely mimic AAA progression, males exhibited pronounced lethality from nicotine in the form of aortic rupture (60%); (3) removal of female sex hormones had minimal effects on nicotine on AngII-induced AAAs; (4) removal of male sex hormones reduced the effects of nicotine on AngII-induced AAAs; (5) sex hormones regulate nicotine metabolism, with the removal of either male or female sex hormones increasing an index of nicotine metabolism; these results suggest that sex hormones (either 17β-estradiol in females or testosterone in males) suppress nicotine metabolism; (6) testosterone, but not 17-β-estradiol, stimulated MMP2 mRNA abundance and reduced markers of a contractile phenotype in murine abdominal aortic VSMC.

We used infusion of AngII to hypercholesterolemic mice as a well-established experimental model to study AAA formation and progression [54–56]. While the AngII model has inherent limitations in directly replicating the human condition, it remains a well-established system to investigate mechanisms underlying AAA development and progression, including the examination of the effects of nicotine [57].

Smoking is one of the largest risk factors for AAA formation, with studies showing that current smokers are more than seven times more likely to have an AAA than age-matched non-smokers [4]. The majority of evidence indicates that nicotine augments AAA formation; however, those with an AAA who continued to smoke exhibited higher growth rates of AAAs and increased propensity for AAA rupture [2,58,59]. Some studies have suggested that smoking is a more significant risk factor for AAA development in women than men [6–8,60]. Unfortunately, even fewer experimental AAA studies have included both sexes in examining influences of smoking and/or nicotine. To directly address interactions between biologic sex and nicotine in AAA development and progression that may guide future studies focused on smoking and human AAAs, this study focused on nicotine’s influence and its interaction with sex hormones on AngII-induced AAAs.

Our findings support the hypothesis that nicotine’s effects on AngII-induced AAAs are regulated by sex hormone influences on nicotine’s pharmacodynamics and/or mechanisms of AAA formation. While co-infusion with AngII + nicotine modestly increased AAA incidence in female mice, the severity and lethality of AAAs were significantly higher in males exposed to nicotine, with 60% of males experiencing aortic rupture. Moreover, GDX of male mice ameliorated effects of nicotine on AngII-induced AAAs, increased an index of nicotine metabolism, and augmented mechanisms of AAA formation (i.e., stimulation of MMP2) in male VSMCs. In contrast, aside from increasing an index of nicotine metabolism, GDX of females did not influence AngII-induced AAAs and had no effects on MMP2 expression in female SMCs as a mechanism of AAA formation. These findings suggest that testosterone, rather than estradiol, plays a more critical role in modulating the synergistic effects of nicotine and AngII on AAA development. The increased lethality from aortic ruptures in males further supports the conclusion that the mechanisms driving nicotine’s effects on AAAs are more active in males, likely due to the testosterone effect in promoting a synthetic VSMC phenotype and MMP2 expression.

It is well recognized that male mice exhibit higher incidences and severity of AngII-induced AAAs compared with females [25,26,31,33], mimicking the human condition. Aside from testosterone [25,31,33] or the removal of a second X chromosome from females [26], very few stimuli have been demonstrated to overcome resistance to AngII-induced AAAs of female mice. While AAA incidences of females in response to AngII in this study were low as reported previously [25,33], co-infusion of nicotine with AngII in females modestly increased AAA incidence by 20–40%. However, nicotine was not able to overcome the resistance of female hypercholesterolemic mice to AngII-induced AAAs. Moreover, in direct comparisons between male and female mice co-infused with nicotine and AngII in the present study, the severity of nicotine’s effects to promote aortic rupture was markedly higher in male than female mice, with very high aortic rupture lethality (60% of mice) upon prolonged nicotine exposures of male mice. To our knowledge, only one previous study included both male and female mice in relation to smoking and AAA development, using daily E-cig vaping with nicotine across two different experimental AAA models (elastase, AngII infusions) [61]. Similar to our findings, previous results demonstrated more pronounced effects of E-cig vaping of nicotine on AngII-induced AAAs in male mice. These results suggest that while nicotine can promote AngII-induced AAA development to some extent in female mice, the severity of nicotine’s effects is more pronounced in males. This more pronounced effect of nicotine on AAA severity in male mice may result from their higher propensity to develop an aneurysm in response to AngII.

To dissect out influences of prolonged nicotine exposure on AAA progression between males and females, in the present study, we increased the duration of exposure to co-infusions of AngII + nicotine (to 56 days) and lowered the AngII infusion dose in males to bring their AAA incidence (67%) closer to females (44%). AAA incidence between females and males co-infused with AngII + nicotine at study endpoint was similar (67%). However, male mice exposed for prolonged durations to AngII + nicotine exhibited pronounced lethality (60% death from aortic rupture), with females exhibiting approximately 30% lethality. Since the quantification of AAA incidence includes mice that succumb from aortic rupture confirmed post-mortem, despite a similar overall AAA incidence (67%) between males and females upon prolonged AngII + nicotine exposures, male mice died from aortic ruptures at approximately double the rate of females. These results suggest that higher AAA prevalence of males in response to AngII infusions does not totally account for the heightened ability of nicotine to promote aortic ruptures of males. Importantly, systolic and diastolic blood pressures, measured during the third week of AngII infusion, were not significantly altered by nicotine co-infusion in either male or female mice. This suggests that the exacerbation of AAA severity and increased aortic rupture incidence in males was independent of any significant changes in systemic blood pressure.

Sex hormones influence experimental AAA formation and severity [24,25,33,34]; however, it remains unclear if sex hormones influence the ability of nicotine to promote AAAs. Previous studies did not identify significant effects of female sex hormones on AngII-induced AAAs [25]. In contrast, testosterone is a strong stimulator of AngII-induced AAAs in both male [24,25,33] and female mice [25,33,34]. In this study, we used GDX in female and male mice to define influences on nicotine-induced regulation of AngII-induced AAAs. Similar to previous findings [25], OVX of female mice did not significantly alter the formation or severity of AngII-induced AAAs. OVX also did not significantly influence AAA development in females co-infused with AngII + nicotine. Paradoxically, rather than promoting the effects of nicotine, OVX modestly reduced the effects of nicotine on the incidence of AngII-induced AAAs. We found that OVX resulted in an increased ratio of 3-trans-hydroxycotinine/cotinine in the sera of females co-infused with AngII + nicotine, indicating enhanced metabolic conversion of nicotine upon the removal of female sex hormones. In agreement with this, higher ratios of trans-3-hydroxycotinine/cotinine have been observed in women compared with men [62]. A twin study using intravenous infusions of nicotine and cotinine demonstrated that clearances were higher in women than men [63]. Similar findings were shown in women taking oral contraceptives, which increased the clearance of both nicotine and cotinine [62]. These findings may result from the effects of 17-β-estradiol to stimulate gene expression of CYP2A6, an enzyme involved in nicotine metabolism [64]. However, results from the present study using OVX to remove female sex hormones suggest the opposite, namely, that female sex hormones (17-β-estradiol and/or progesterone) suppress nicotine metabolism in mice. Interestingly, previous studies demonstrated that OVX of female rats reduced mean plasma nicotine concentrations following intravenous nicotine administration [65], supporting higher nicotine metabolism in the absence of female sex hormones as observed in the present study. Nevertheless, results from this study do not support profound female sex hormone regulation of nicotine’s influences on AAA formation and severity in females.

In contrast, we found that total GDX of male mice reduced the effects of nicotine to augment the formation and severity of AngII-induced AAAs. These results are in agreement with previous studies demonstrating that male androgen promotes the formation and severity of AngII-induced AAAs [24,25,33,66]. In this study, the number of male testes was inversely related to the ability of nicotine to promote the formation and severity of AngII-induced AAAs. Since male sex hormones are known to influence the ability of AngII to promote AAA formation [24,25,33], in this study, it is difficult to discern whether testosterone is a specific regulator of nicotine’s influences on AngII-induced AAAs. Moreover, since the sera ratios of trans-3-hydroxycotinine/cotinine increased in GDX male mice, it is possible that increased rates of nicotine metabolism contributed to lower AAA formation and severity in GDX males.

Our results support a potential role for sex hormone regulation of aortic VSMC MMP2 as a mechanism by which nicotine promotes the formation and severity of AngII-induced AAAs in male mice. The inclusion of MMP2 as a mechanistic focus was based on its established role in aortic aneurysms [67], despite the limitations of clinical trials targeting broad MMP inhibition to treat AAAs. Abdominal aortas from male mice co-infused with AngII + nicotine exhibited higher mRNA abundance of MMP2, and prolonged exposures to nicotine in AngII-infused male mice resulted in a significantly greater numbers of breaks in medial elastin. Previous studies demonstrated that MMP2 immunostaining in AAA tissue sections of mice co-infused with AngII and nicotine was reduced by the administration of a JNK inhibitor [22]. Additionally, studies have implicated the activation of MMPs in response to nicotine in several cell types and animal models [17]. Our results extend previous findings by demonstrating that testosterone increased MMP2 mRNA abundance in abdominal aortic VSMCs from male mice. Moreover, testosterone exposures in abdominal aortic VSMCs decreased markers of a contractile SMC phenotype, supporting a switch to a more synthetic SMC phenotype that has been linked to AAA development [48]. Interestingly, previous results demonstrated that nicotine exposures promoted a synthetic phenotype in human vascular SMC [68]. Our results suggest a positive interaction between testosterone and nicotine to promote a synthetic SMC phenotype associated with stimulated MMP2. The interplay between testosterone and nicotine may contribute to more pronounced AAA formation and severity in male mice.

Results from this study have limitations that warrant further investigation. First, the low level of AngII-induced AAAs in female mice could affect the power to detect statistical differences in parameters such as abdominal aortic lumen diameter. Additionally, this highlights a broader limitation in statistical power for detecting sex differences due to lower AAA incidences of female AngII-infused mice. Future studies could address this by employing larger human cohorts or alternative experimental models that increase AAA incidence of females. Second, while we identified sex-specific effects of nicotine on AAA formation, the mechanistic pathways underlying these differences remain to be fully elucidated. Specifically, further studies are needed defining the causal relationship between MMP-2 expression in VSMCs, sex hormones, and aneurysm formation. Finally, translating findings from models to humans will require validation in clinical studies, given potential differences in AAA pathophysiology between species.

In summary, results from this study demonstrate that nicotine markedly increases aortic rupture and lethality in male mice, with modest influences in females. The removal of male sex hormones attenuated the effects of nicotine to promote AngII-induced AAAs, while the removal of female sex hormones had modest effects. The interplay between testosterone and nicotine in promoting a synthetic SMC phenotype with increased MMP2 expression may contribute to the more pronounced influence of nicotine on AAAs in males.

**Clinical perspective**

- Smoking is an irrefutable risk factor for abdominal aortic aneurysms (AAAs), with evidence suggesting that women may be at a higher risk than men. In this study, we explored the effects of nicotine on angiotensin II-induced AAAs in male and female mice to decipher the sex-specific mechanisms behind AAAs development that are influenced by smoking.
- Nicotine exacerbated aortic rupture and matrix metalloproteinase 2 expression in males. Gonadectomy reduced AAAs severity in males but did not significantly affect females. Testosterone changed the phenotype of abdominal aortic smooth muscle cells (SMCs) from males, while estrogen showed minimal effect on female SMCs.
- Understanding the interaction between sex hormones and nicotine metabolism in influencing AAA risk offers insights for developing targeted therapies for smoking-related vascular diseases in men and women.

## Supplementary material

Online supplementary figures

## Data Availability

The data supporting this study’s findings are available from the corresponding author upon reasonable request.

## Abbreviations

AAAs: abdominal aortic aneurysms
AngII: angiotensin II
GDX: gonadectomy
LC-MS/MS: liquid chromatography-tandem mass spectrometry
MMP2: matrix metalloproteinase 2
OVX: ovariectomy
SMCs: smooth muscle cells
SRM: selected reaction monitoring
VSMCs: vascular smooth muscle cells
