# Supplementary material for: Nicotine promotes AngII-induced abdominal aortic aortopathies in female and male mice: role of sex hormones
Source: Clin Sci (Lond). 2025 Apr 23;139(8):411–29. doi: 10.1042/CS20255689 (PMC12204014; doi:10.1042/CS20255689)
Supplement: Online supplementary figures [file CS-139-08-CS20255689-s001.pdf]

**Supplemental Figure I**. Release of nicotine from Alzet osmotic minipumps (model 1004, release rate of 0.13 µl/hr) over 28 days. Pumps contained nicotine (35.8 mg/ml, analogous to dose of 4 mg/kg/day) and AngII (13 mg/ml, analogous to 1,000 ng/kg/min) dissolved in 0.9% sterile saline. Dissolved solutions were filtered (0.22 µm) and used to fill three pumps incubated at 37°C for 28 days. On days 7, 14 and 21 saline was replaced fresh for each pump. Nicotine was measured by isocratic HPLC using a C18 reverse-phase column (ACE Equivalence C18 column, 4.6mm x 15cm) with UK detection (254 nm). Concentrations of nicotine in samples were calculated from internal nicotine standards of known concentrations. Using the minipump release rate, nicotine release rates for each 7 day period were estimated to be 390 µg/ml.

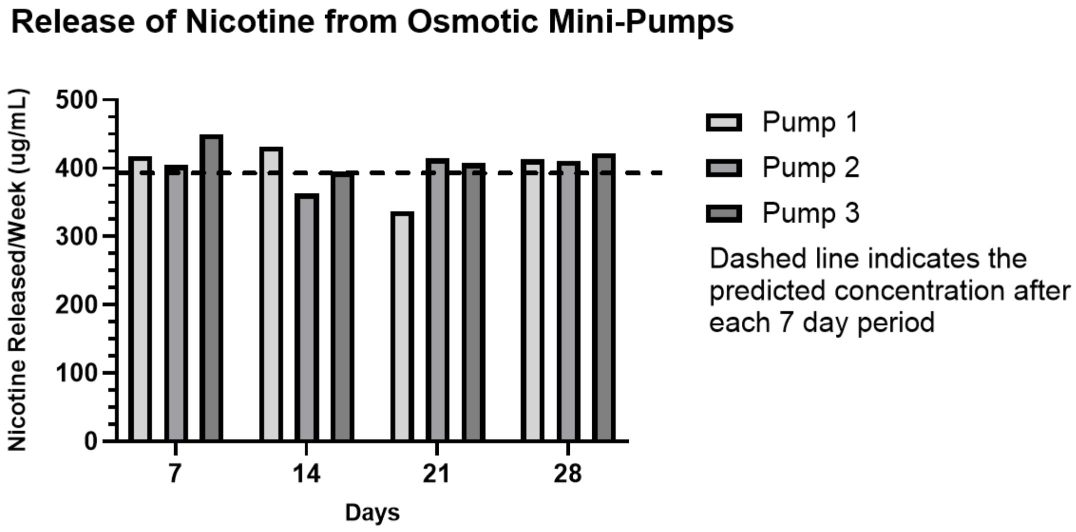

**Supplemental Figure II.** Systolic and diastolic blood pressures of male and female mice infused with AngII+/- nicotine for 21 days. Symbols are individual values, while bars are mean $\pm$ SEM from N= 6-10 mice/group/treatment.

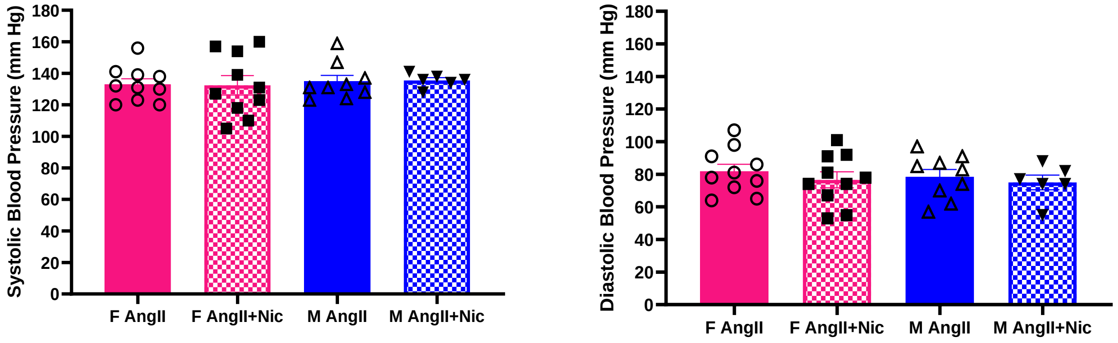

**Supplemental Figure III.** Elastin degradation within abdominal aortic sections of male mice infused with AngII +/- nicotine for 56 days. Symbols are individual values, while bars are mean ± SEM from N=6 aortic sections/ treatment. *, P<0.05 compared to AngII only.

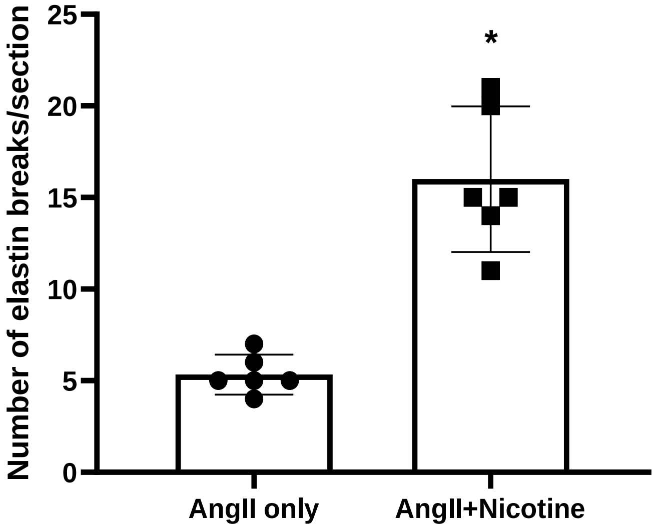

**Supplemental Figure IV.** Sera concentrations of trans-3-hydroxycotinine for mice in various studies. A, Female and male mice infused with AngII+/- nicotine for 56 days. B, Female sham or ovariectomized (OVX) mice infused with AngII+/- nicotine for 28 days. C, Male mice with two, one or no testes infused with AngII plus nicotine for 28 days. Individual mice are represented by symbols within each bar, with mean ± SEM from n = 5-15 mice/group. *, P<0.05 compared to AngII within sex. **, P<0.05 compared to male within treatment.

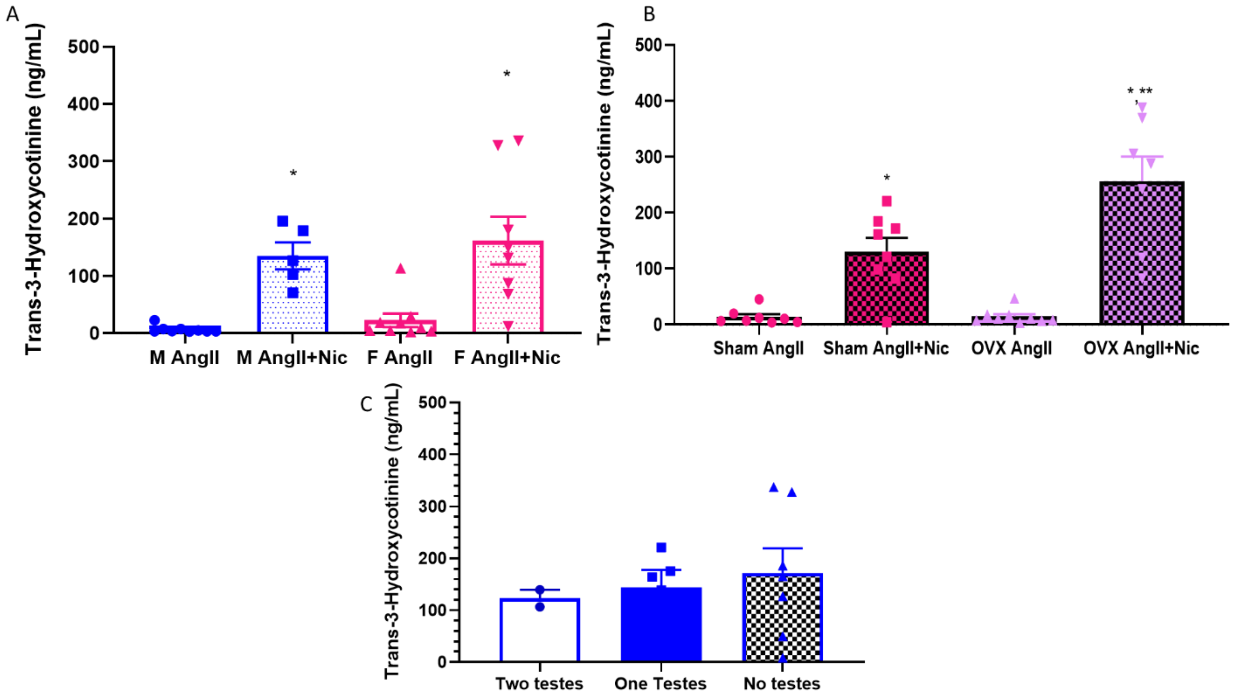

**Supplemental Figure V.** Sex hormone regulation of α-2nAchR mRNA abundance in abdominal aortic VSMC. Abdominal aortic female and male VSMC were treated with 17-β-estradiol or testosterone, respectively. Symbols individual values, while bars are mean + SEM from N=3/group/treatment.

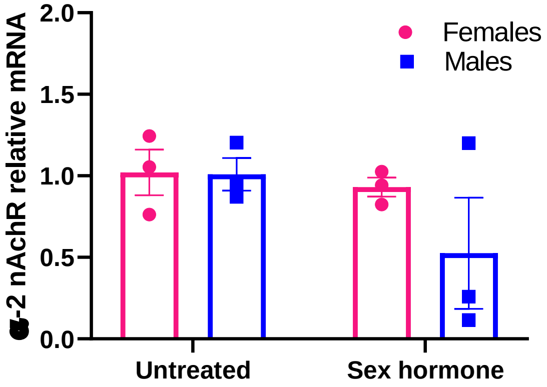

**Supplemental Table I. MS transitions used to quantify nicotine and metabolites.**

| Compound | Retention Time (min) | RT Window (min) | Precursor (m/z) | Product (m/z) | Collision Energy (V) |
|---|---|---|---|---|---|
| trans-3-hydroxycotinine | 1.1 | 1 | 192.9 | 80 | 30 |
| trans-3-hydroxycotinine | 1.1 | 1 | 192.9 | 106 | 29 |
| trans-3-hydroxycotinine | 1.1 | 1 | 192.9 | 134 | 20 |
| cotinine | 1.8 | 1 | 177 | 80 | 26 |
| cotinine | 1.8 | 1 | 177 | 98 | 22 |
| cotinine | 1.8 | 1 | 177 | 146 | 18 |
| cotinine-d3 | 1.8 | 1 | 180.1 | 80 | 27 |
| cotinine-d3 | 1.8 | 1 | 180.1 | 101 | 24 |
| cotinine-d3 | 1.8 | 1 | 180.1 | 138.8 | 13 |
| nicotine | 2.4 | 1 | 163.1 | 117 | 28 |
| nicotine | 2.4 | 1 | 163.1 | 130 | 22 |
| nicotine | 2.4 | 1 | 163.1 | 132 | 16 |
| nicotine-d4 | 2.4 | 1 | 167.2 | 121 | 28 |
| nicotine-d4 | 2.4 | 1 | 167.2 | 134 | 22 |
| nicotine-d4 | 2.4 | 1 | 167.2 | 136 | 16 |
